# Soy Infant Formula and Seizures in Children with Autism: A Retrospective Study

**DOI:** 10.1371/journal.pone.0080488

**Published:** 2014-03-12

**Authors:** Cara J. Westmark

**Affiliations:** Department of Neurology, University of Wisconsin, Madison, Wisconsin, United States of America; Nathan Kline Institute and New York University School of Medicine, United States of America

## Abstract

Seizures are a common phenotype in many neurodevelopmental disorders including fragile X syndrome, Down syndrome and autism. We hypothesized that phytoestrogens in soy-based infant formula were contributing to lower seizure threshold in these disorders. Herein, we evaluated the dependence of seizure incidence on infant formula in a population of autistic children. Medical record data were obtained on 1,949 autistic children from the SFARI Simplex Collection. An autism diagnosis was determined by scores on the ADI-R and ADOS exams. The database included data on infant formula use, seizure incidence, the specific type of seizure exhibited and IQ. Soy-based formula was utilized in 17.5% of the study population. Females comprised 13.4% of the subjects. There was a 2.6-fold higher rate of febrile seizures [4.2% versus 1.6%, OR = 2.6, 95% CI = 1.3–5.3], a 2.1-fold higher rate of epilepsy comorbidity [3.6% versus 1.7%, OR = 2.2, 95% CI = 1.1–4.7] and a 4-fold higher rate of simple partial seizures [1.2% versus 0.3%, OR = 4.8, 95% CI = 1.0–23] in the autistic children fed soy-based formula. No statistically significant associations were found with other outcomes including: IQ, age of seizure onset, infantile spasms and atonic, generalized tonic clonic, absence and complex partial seizures. Limitations of the study included: infant formula and seizure data were based on parental recall, there were significantly less female subjects, and there was lack of data regarding critical confounders such as the reasons the subjects used soy formula, age at which soy formula was initiated and the length of time on soy formula. Despite these limitations, our results suggest that the use of soy-based infant formula may be associated with febrile seizures in both genders and with a diagnosis of epilepsy in males in autistic children. Given the lack of data on critical confounders and the retrospective nature of the study, a prospective study is required to confirm the association.

## Introduction

Autism is a cluster of complex neurobiological disorders known as autism spectrum disorders. The core features include impairments in social interaction and communication, and repetitive stereotyped behavior. Autism is estimated to occur in 1 in 88 children with prevalence 4.7-fold higher in males [1]. The etiology of autism is not known but genetic as well as environmental factors likely affect the severity of symptoms [2]–[4]. Our findings in rodent models of neurological disease demonstrate that soy ingestion during a critical period of sensory development significantly increases seizure activity [5]. Mouse colonies are typically maintained on Purina-type lab chows, which are grain-based with their protein content derived from soybeans [6]. Soybeans are rich in phytoestrogens, “plant estrogens”, which are transferred to offspring through the placenta as well as maternal milk [7]. The steady-state level of phytoestrogens found in serum (2,338±531 ng/mL) of Purina-fed mice is greater than endogenous estrogen levels by at least 30,000-fold and has immense potential to affect development [8]. The soy phytoestrogen daidzein was identified as a seizure-promoting ingredient in mice [5]. Thus, we hypothesized that the use of soy-based infant formulas could be contributing to seizure incidence in autism and other neurodevelopmental disorders.

While there are many health benefits for adults that are associated with the consumption of soy products in terms of prevention of age-related cardiovascular disease and osteoporosis [9], [10], there is a paucity of studies on the effects of phytoestrogens on fetal and early childhood development [11]–[16]. Nearly a quarter of infant formulas are based on soy protein and have high phytoestrogen levels approaching 4.5–8 mg/kg/day [17], [18]. Considering body weight, these infants are getting six to 11 times the dose of phytoestrogens necessary to exert hormone-like effects in adults [17]. In placental mammals, the fetus is continuously exposed to high levels of estrogen from the placenta and the mother. Studies in pregnant rats demonstrate that the placenta acts as a sink for phytoestrogens, and that while transport of phytoestrogens across the placenta is inefficient, low levels are found in the fetus and are sufficient for activation of estrogen receptor beta [19]. Thus, environmental exposure to phytoestrogens or synthetic estrogens during gestation and/or postnatal development could disrupt the function of the natural steroid hormones and contribute to the incidence of idiopathic disorders such as autism. The aim of this retrospective study was to determine if there was an association between seizure incidence and the use of soy-based infant formula by utilizing medical record data available through the Simons Foundation Autism Research Initiative (SFARI) Simplex Collection, which is a medical record database and biological specimen resource for the identification of genetic risk factors for autism. We present data below suggesting a potential association between the use of soy-based infant formula and increased incidence of febrile seizures, simple partial seizures and epilepsy in autistic children.

## Results

### Demographics

The population for this study was defined as all probands in the Simons Simplex Collection who had non-null medical record data for the type of infant formula used. Demographics indicate that there were 86.6% males and 13.4% females in a population size of 1,949 autistic children. The 6.5-fold increase in the number of male subjects is supported by epidemiological data indicating that autism is 4.7-fold more prevalent in boys than girls [1]. Soy-based infant formula was used by 17.6% of the male and 17% of the female probands (Table 1). The cohorts have similar population characteristics regarding ethnicity, age at evaluation, birth weight, and scores on the ABC, ADI-R and ADOS.

**Table 1:** Characteristics of the Autistic Population.

		Soy Formula	Non-Soy Formula
**Population Size, % (N)**	**Male**	17.6 (297)	82.4 (1391)
	**Female**	17 (44)	83.1 (217)
**Ethnicity Males, %**	**African American**	5.1	3.4
	**Asian**	2.4	4.2
	**Other** a	13.5	13.8
	**White**	79.1	78.6
**Ethnicity Females, %**	**African American**	9	3.2
	**Asian**	5	3.2
	**Other** a	9	11.1
	**White**	77	82.5
**Age at Evaluation, Years (SD)**	**Male**	9.2 (3.8)	8.9 (3.6)
	**Female**	8.3 (3.4)	9.0 (3.7)
**Birth Weight, Pounds (SD)**	**Male**	7.1 (1.2)	7.3 (1.2)
	**Female**	6.7 (1.1)	7.0 (1.2)
**ABC Score, Mean (SD)**	**Male**	48.3 (25.4)	46.2 (26.2)
	**Female**	49.0 (28.0)	46.1 (24.9)
**ADI-R Score** b **, Mean (SD)**	**Male**	36.6 (8.9)	35.9 (9.3)
	**Female**	36.5 (9.7)	36.1 (10.0)
**ADOS Score** c **, Mean (SD)**	**Male**	28.2 (8.9)	28.4 (9.0)
	**Female**	28.8 (8.5)	29.3 (9.8)

a Other in the Ethnicity category includes other, not specified, more than one race, Native American, and Native Hawaiian.

b ADI-R total score is the sum of the Reciprocal Social Interaction, Nonverbal and Repetitive Behavior/Stereotyped Patterns scores. The Verbal score was omitted in order to include both verbal and nonverbal participants.

c ADOS total score is the sum of the Communication and Social Interaction, Restricted and Repetitive Behavior, and Social Affect scores.

### Seizure and epilepsy rates

There was a 2.6-fold higher rate of febrile seizures in the soy-fed cohort (4.2% seizures with soy and 1.6% seizures without soy) (odds ratio = 2.6, 95% confidence interval 1.3–5.3; *P* = 0.003) (Table 2). The results were statistically significant for females (9% seizures with soy and 1.4% seizures without soy) (odds ratio = 7.2, 95% confidence interval 1.3–42; *P* = 0.02) with a Bonferroni correction for gender comparisons, but were not statistically significant in males (3.4% seizures with soy and 1.7% seizures without soy) (odds ratio = 2.1, 95% confidence interval 0.92–4.7; *P* = 0.050). The prevalence of childhood epilepsy in the general population is 0.63% [21]. In this autism cohort, epilepsy prevalence ranged from 1.6–3.8% dependent on gender and diet (Table 2). A comorbid diagnosis of autism and epilepsy was more prevalent in males fed soy-based formula (odds ratio = 2.4, 95% confidence interval 1.1–5.2; *P* = 0.02) than females (odds ratio = 1.4, 95% confidence interval 0.056–14; *P* = 0.8). Febrile seizures are associated with an increased incidence of epilepsy [22]. In this study population, there was no overlap between subjects that reported epilepsy and febrile seizures. However, there were only 39 subjects reporting febrile seizures. Epilepsy developed by seven years of age in 2% of children who had experienced at least one febrile seizure [22]; thus, we would predict less than one subject in our study population with both epilepsy and febrile seizures.

**Table 2:** Prevalence of Seizures in Autistic Subjects Dependent on Soy Formula.

	Male		Female		Combined	
Phenotype, % (N)	Soy	Non-Soy	Soy	Non-Soy	Soy	Non-Soy
**Febrile Seizures**	3.4^a^ (290)	1.7 (1368)	9^b^ (43)	1.4 (213)	4.2^c^ (333)	1.6 (1581)
**Epilepsy Diagnosis**	3.8^d^ (291)	1.6 (1352)	3 (39)	1.9 (211)	3.6^e^ (330)	1.7 (1563)
**Infantile Spasms**	0.7 (292)	0.1 (1377)	0 (42)	0 (215)	0.6 (334)	0.1 (1592)
**Atonic Seizures**	0.3 (291)	0.2 (1377)	0 (42)	0 (215)	0.3 (333)	0.1 (1592)
**Tonic Clonic Seizures**	1.4 (291)	1.7 (1378)	0 (42)	3.7 (216)	1.2 (333)	1.9 (1594)
**Absence Seizures**	3.4 (291)	2.0 (1378)	2 (42)	2.3 (216)	3.3 (333)	2.0 (1594)
**Simple Partial Seizures**	1.0 (292)	0.3 (1377)	2 (42)	0 (215)	1.2^f^ (334)	0.3 (1592)
**Complex Partial Seizures**	0.3 (291)	0.4 (1377)	2 (42)	0.4 (215)	0.6 (333)	0.4 (1592)

N = number of subjects in cohort.

a *P* = 0.050 as determined by the Pearson's uncorrected chi-squared test, OR = 2.1, 95% CI = 0.92–4.7.

b *P* = 0.02 as determined by Fisher's exact test (two-tail), OR = 7.2, 95% CI = 1.3–42.

c *P* = 0.003 as determined by the Pearson's uncorrected chi-squared test, OR = 2.6, 95% CI = 1.3–5.3.

d *P* = 0.02 as determined by the Pearson's uncorrected chi-squared test, OR = 2.4, 95% CI = 1.1–5.2.

e *P* = 0.02 as determined by the Pearson's uncorrected chi-squared test, OR = 2.2, 95% CI = 1.1–4.7.

f *P* = 0.04 as determined by Fisher's exact test (two-tail), OR = 4.8, 95% CI = 1.0–23.

The soy-based formula was not associated with statistically significant higher rates of infantile spasms, atonic (drop attack), generalized tonic clonic (grand mal), absence (petit mal), or complex partial seizures for either gender albeit many of these seizure types were very infrequent (Table 2). Although not statistically significant, infantile spasms (three cases total) were only observed in the males and were higher in soy-fed infants (odds ratio = 9.5, 95% confidence interval 0.68–265; *P* = 0.08). Simple partial seizures (eight cases total) were higher with soy-based formula for both genders but only reached statistical significance when combining the data (odds ratio = 4.8, 95% confidence interval 1.0–23; *P* = 0.04). Thus, a larger study population is necessary to confirm whether the use of soy-based infant formula is associated with higher rates of infantile spasms and simple partial seizures in both boy and girls with autism. The age of seizure onset (Table 3) and IQ (Table 4) were not statistically different dependent on infant formula (Student T-test, *P* = 0.9 for soy versus non-soy in both males and females for age of onset of febrile seizures; *P* = 0.3 for soy versus non-soy in both males and females for age of onset of non-febrile seizures; *P* = 0.4–0.5 for soy versus non-soy in both males and females for full-scale IQ and non-verbal IQ; *P* = 0.6–0.8 for soy versus non-soy in males and females for verbal IQ). In summary, our results suggest that soy-based infant formula may be associated with higher rates of febrile seizures, epilepsy and simple partial seizures in autistic children.

**Table 3:** Age of Seizure Onset with Soy-Based Formula.

		Febrile Seizures			Non-Febrile Seizures		
		Years	SD	N	Years	SD	N
**Males**	**Soy**	2.0	1.5	16	3.6	2.5	22
	**Non-Soy**	2.0	1.5	43	4.4	3.3	71
**Females**	**Soy**	2.0	1.7	5	2.1	1.3	3
	**Non-Soy**	1.9	1.1	7	4.0	3.0	11

**Table 4:** IQ with Soy-Based Formula.

		Full-Scale IQ			Non-Verbal IQ			Verbal IQ		
		Mean	SD	N	Mean	SD	N	Mean	SD	N
**Males**	**Soy**	80.1	27.8	293	83.3	25.8	294	77.1	31.6	294
	**Non-Soy**	81.5	28.1	1364	84.8	26.4	1366	78.2	31.3	1366
**Females**	**Soy**	72.0	28.7	41	74.8	25.1	41	72.3	35.0	41
	**Non-Soy**	75.5	27.7	212	78.2	26.1	212	74.1	31.2	212

## Discussion

In this autism-based population study, febrile seizures were 2.6-fold more prevalent in children fed soy-based formula. Febrile seizures are convulsions brought on by a fever in infants or small children. They are the most common type of childhood convulsive events occurring in 2–5% of children in Europe and North America and in 6–9% in Japan [23], an Asian country with a soy-rich diet. A diagnosis of epilepsy was also significantly elevated in association with the consumption of soy. The prevalence of epilepsy in autism is 21.4% in subjects with intellectual disability and 8% in subjects without intellectual disability [24]. Our study indicates a comorbidity of autism and epilepsy of 1.6–3.8% dependent on gender and diet. This is significantly higher than the general population, but lower than published reports of epilepsy in autism likely due to the inclusion criteria of probands with relatively little intellectual disability. The 2-fold difference in epilepsy comorbidity between soy (3.6%) and non-soy (1.7%) groups was statistically significant (P = 0.02). The 2.6-fold difference in febrile seizure rates between soy (4.2%) and non-soy (1.6%) cohorts was highly statistically significant (P = 0.003). These may be considered by some readers as small percentages in each group who had seizures, but the two-fold or greater differences between soy and non-soy diets are statistically significant. Pharmaceutical interventions that reduced the incidence of febrile seizures or epilepsy by 2-fold would be in demand, and we consider a possible dietary intervention equally relevant.

Seizures are a serious and common phenotype in many neurological disorders besides autism and epilepsy including fragile X syndrome [25], Alzheimer's disease [26], Down syndrome [27], tuberous sclerosis [28] and Rett syndrome [29]. According to the Epilepsy Foundation, ten percent of the American population will experience a seizure in their lifetime; however, the underlying molecular mechanisms that cause seizures are not well understood. Seizure severity is likely a combination of both genetic and environmental factors. Our hypothesis is that the effects of an underlying genetic mutation that lowers seizure threshold may be exacerbated, for example, by dietary exposure to high concentrations of phytoestrogens. This would be particularly significant for individuals whose diet is solely soy-based, for example, soy-based infant and gastric tube feeding formulas. Nearly a quarter of infant formulas are soy-based; yet, there is no epidemiological data examining the incidence of seizures, autism, or the severity of fragile X syndrome and other developmental disorders in children fed soy-based versus non-soy-based formula.

The strength of this study design includes a large sample size of autistic children with comprehensive medical record histories. An autism diagnosis was based on ADI-R and ADOS scores. The limitations of the study include: (1) it is based on retrospective data regarding the type of formula, (2) the quality of the epilepsy data is declarative, (3) there is low statistical power regarding the gender comparison analyses, and (4) there is lack of data regarding confounding issues. Data regarding the type of formula consumed was based on parental recall, which is expected to decrease in accuracy as the length of recall increases. A diagnosis of epilepsy was defined as either a specific report of epilepsy on the ADI-R or at least two seizures on the medical history report, which are also based on parental recall. This study is a retrospective analysis of an existing data set and a neurologist did not verify the diagnoses. However, according to NINDS, detailed medical history reports of seizure history are considered one of the best methods available to determine if a person has epilepsy and what kind of seizures he/she has (http://www.ninds.nih.gov/disorders/epilepsy/detail_epilepsy.htm#242093109). There were significantly less female subjects than males in accordance with current autism prevalence statistics. Despite this limitation, our results suggest that the use of soy-based infant formula may be associated with febrile seizures in females and with epilepsy in males. A larger female cohort is necessary to confirm whether the use of soy-based infant formula may be associated with epilepsy comorbidity in girls with autism. Data regarding critical confounders such as the reasons the subjects used soy formula, age at which soy formula was initiated and the length of time on soy formula are not available. It is possible that sick children who were fed soy-based formula for various diagnosed or undiagnosed health complications are somehow predisposed to seizures; thus, soy could be a surrogate marker for an underlying condition that lowers seizure threshold. If soy is the causative agent in seizure induction, it would be expected that infants fed soy formula at younger ages and/or for longer periods of time would exhibit an increased incidence of seizures. A prospective study will be required to address these confounding issues. A possible criticism is that subjects, who were fed soy-based infant formula because they were allergic to cow's milk, had allergies that made them vulnerable to illnesses associated with fever-induced convulsions. Though the retrospective nature of the data does not allow us to make definitive conclusions regarding this point, we found that 2.7% of females and 1.7% of males in the study population reported allergies, but no subjects reported both allergies and febrile seizures suggesting that this criticism may not be valid.

We found that the use of soy-based formula was a potential risk factor for increased seizure incidence and epilepsy in autism. Independent studies in other infant populations (typically developing, fragile X and Down syndrome) are necessary before a general association between increased seizure risk and soy-based formula can be established. Of note, the SFARI autism database is not representative of all ASD because of exclusion criteria that bar important genetic causes of both seizures and ASD. For example, fragile X syndrome is the leading genetic cause of autism and accounts for approximately 5% of autism cases [30]. The studied population exhibited moderate to severe autistic symptoms with relatively little intellectual disability. The excluded population would be expected to be much lower functioning and perhaps more affected by environmental factors such as soy phytoestrogens. We did not observe statistically significant differences in the age of seizure onset or IQ with the use of soy-based infant formula, albeit there was a trend for a younger age of non-febrile seizure onset and lower IQ. The mean age of non-febrile seizure onset was 2.1–4.4 years dependent on gender and diet. These data raise the questions - can soy ingestion during infancy alter brain development such that seizure threshold, IQ and/or behavior are affected later in life? The data collection strategy for the SFARI Simplex Collection was not designed to answer these types of questions. The findings described herein are suggestive of a link between soy exposure and seizures, and prospective evaluation is needed.

## Methods

### Ethics statement

The institutional review protocol governing the Simons Simplex Collection was approved by the Institutional Review Board at Columbia University Medical Center. Written informed consent was provided by all guardians or research subjects. The privacy of participants was protected by using global unique identifiers. The research protocol for using the Simons Simplex Collection in this study was approved by the Human Research Protection Program at the University of Wisconsin-Madison, which determined that the study qualified for exemption.

### Participants

SFARI in collaboration with medical centers across North America collected high quality phenotype data and biospecimens from 2,644 autism simplex families beginning in 2008. A simplex family is one in which only one child (the proband) is on the autism spectrum, while both biological parents and all siblings are not. All collection sites used the same inclusion and exclusion criteria, administered the same instruments and followed the same protocols in collecting biospecimens. Families were recruited from a coalition of clinics located at Baylor College of Medicine, Children's Hospital of Boston, Columbia University, Emory University, McGill University, University of California-Los Angeles, University of Illinois at Chicago, University of Michigan, University of Missouri, University of Washington, Vanderbilt University and Yale University.

The inclusion criteria included proband age and a diagnosis of autism spectrum disorder. The proband in the family was between four years and 17 years and 11 months of age when the phenotype measures were administered and the data collected. On the Autism Diagnostic Interview-Revised (ADI-R), the proband was required to meet one of the following criteria: (1) standard cutoff on the social and communication domains, (2) standard cutoff on the social domain and within two points of communication cutoff, (3) standard cutoff of the communication domain and within two points of social cutoff, or (4) within one point of the standard cutoffs for both the social and communication domains. On the Autism Diagnostic Observation Schedule (ADOS), the proband must have received a valid and reliable administration and must have met the cutoffs for autism spectrum disorders or autism. On the Mullen Scales of Early Learning, the Differential Ability Scales-II, the Wechsler Intelligence Scale for Children-IV or the Wechsler Abbreviated Scale of Intelligence, the proband must have had a nonverbal deviation or ratio IQ score greater than or equal to 60 (four years of age) or greater than or equal to 40 (between five and eight years of age). Participants eight years of age or older must have had a nonverbal mental age of 36 months or older. The proband was also required to have a clinical “Best Estimate Diagnosis” of autistic disorder, Asperger's disorder, or pervasive developmental disorder not otherwise specified made by a psychologist or physician.

The exclusion criteria included: (1) pregnancy and birth issues for probands including fewer than 36 weeks gestation and less than 2,000 grams at birth, or a history of maternal pregnancy or birth complications; (2) other disorders or limitations in the proband including a positive diagnosis for fragile X syndrome or Down syndrome, sensory or motor difficulties that would preclude valid use of diagnostic instruments, or a history of severe nutritional or psychological deprivation; (3) sibling diagnosed with an autism spectrum disorder, mental retardation (except Down syndrome), schizophrenia, or a psychiatric disorder requiring treatment with more than one psychotropic medication; (4) sibling with an Adaptive Behavior Standard score on the Vineland-II that was 70 or below or an Individualized Education Plan for extensive special education services; (5) parent diagnosed with an autism spectrum disorder, mental retardation, or schizophrenia; or (6) any second- or third-degree relative diagnosed with an autism spectrum disorder

Simons Simplex Collection: Medical record data for the autistic probands and family members were available through an interactive database that facilitated correlations between clinical, genetic, and neurobiological data [20]. The dataset utilized for this study was from the Simons Simplex Complex version 14 Public Cohort, released March 21, 2012 (http://sfari.org/resources/sfari-base). The proband study participants exhibited moderate to severe autistic symptoms with relatively little intellectual disability. Data regarding the use of soy-based infant formula and the type of seizures were obtained from the medical history form, a questionnaire regarding the proband and administered to the parent by the clinical research staff. Specific questions regarding seizures included: (1) Has the proband ever been diagnosed with febrile seizures (no, yes, not sure)? (2) What was the age of diagnoses/onset (years or months)? (3) Has the proband ever been diagnosed with seizures (no, yes, not sure)? If Yes, (4) what was the age of onset (years, months, not sure), type (grand mal/generalized tonic clonic, petit mal/absence, infantile spasms, atonic/drop attacks, complex partial, simple partial/focal), frequency (times per day, week, month or year) and date of last seizure? A diagnosis of epilepsy was defined as either a specific report of epilepsy on the ADI-R or at least 2 seizures on the medical history form.

### Data Coding & Analyses

The febrile and non-febrile seizure variables in the SFARI database combined information about the presence of seizures from the ADI-R (question 85) and the medical history form. In the database the variables were labeled “Febrile Seizures :: Proband CDV” and “Non Febrile Seizures :: Proband CDV”, respectively. Coding of the febrile seizure data included scores of 0, 1 and 2 where 0 indicated there was no evidence of the presence of febrile seizures, 1 indicated the possible presence of febrile seizures or the caregiver reported that they were “not sure” if the child had experienced febrile seizures, and 2 indicated the presence of febrile seizures reported. The non-febrile seizure data coding included scores of 0, 1, 2 and 3 where 0 indicated that there was no evidence for the presence of non-febrile seizures, 1 indicated the possible presence of non-febrile seizures or the caregiver report that they were “not sure” if the child had experienced non-febrile seizures, 2 indicated the likely presence of non-febrile seizures and 3 indicated that a diagnosis of epilepsy was reported. We did not include subjects with scores of 1 in our analysis. Scores of 0 were counted as seizure negative and scores of 2 or 3 were counted as seizure positive.

Data were analyzed in accordance with STROBE guidelines (Figure S1). Percentages, means, standard deviations and 95% confidence intervals were computed to describe the population. To statistically test for differences in seizure incidence across groups, Pearson's uncorrected chi-squared approximation and Fisher's exact test (two-tail) were used. The Pearson's uncorrected chi-squared approximation was only utilized in cases where there were at least five expected outcomes per cell. Statistical significance for age of seizure onset and IQ were determined by Student t-test analyses. Statistical significance was defined as *P*<0.050. For multiple comparisons (male versus female), a Bonferroni correction was applied and statistical significance was defined as P<0.025. The number of subjects at each stage of the study is reported in the corresponding tables (Tables 1–4) as well as in a flow chart (Figure S2).

## Conclusions

Published estimates of formula intolerance range from 2%–7.5% [31]; yet, nearly a quarter of infant formulas are soy-based suggesting that they are used excessively. The current position of the American Academy of Pediatrics is, “There is no conclusive evidence from animal, adult human, or infant populations that dietary soy isoflavones may adversely affect human development, reproduction, or endocrine function [32].” The data presented herein provide evidence that the use of soy-based formula may be associated with reduced seizure threshold in a vulnerable population of children diagnosed with autism. Given the lack of data on critical confounders in this retrospective study, a prospective study is required to confirm this association.

## Supporting Information

Figure S1 **Completed checklist of items included in reports of observational studies as recommended by STROBE guidelines and PLOS ONE editorial staff.**
(PDF)

Figure S2 **Flowchart of SFARI subject numbers per analysis as recommended by STROBE guidelines and PLOS ONE editorial staff.**
(JPG)
