# Supplementary figures and images for: Soy Infant Formula and Seizures in Children with Autism: A Retrospective Study
Source: PLoS One. 2014 Mar 12;9(3):e80488. doi: 10.1371/journal.pone.0080488 (PMC3951190; doi:10.1371/journal.pone.0080488)

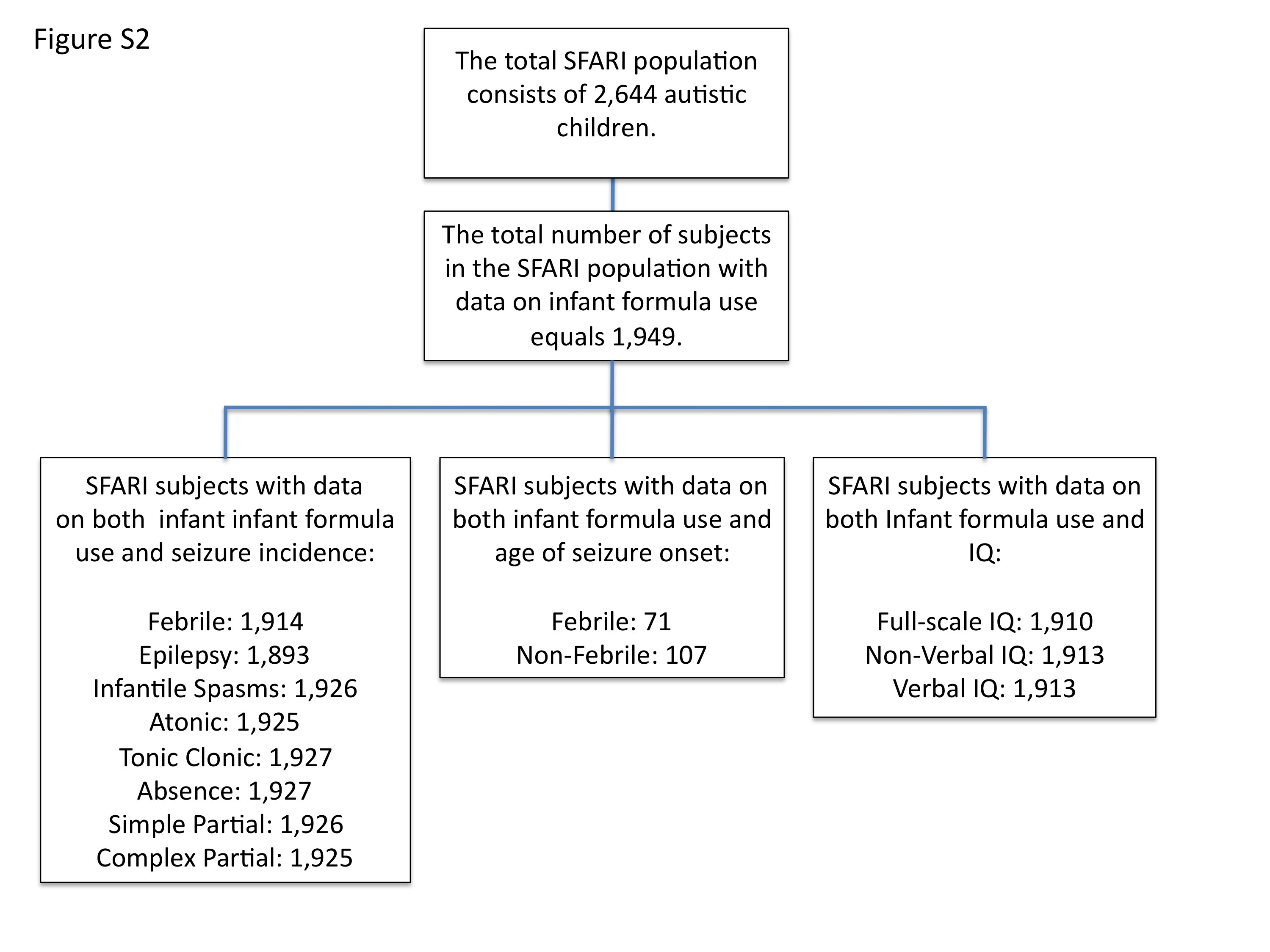

Supplement: Figure S2 — Flowchart of SFARI subject numbers per analysis as recommended by STROBE guidelines and PLOS ONE editorial staff. (JPG) [file pone.0080488.s002.jpg]
